# Generalizations of Talagrand Inequality for Sinkhorn Distance Using Entropy Power Inequality †

**DOI:** 10.3390/e24020306

**Published:** 2022-02-21

**Authors:** Shuchan Wang, Photios A. Stavrou, Mikael Skoglund

**Affiliations:** 1 Communication Systems Department, EURECOM, 06904 Sophia Antipolis, France; shuchan.wang@eurecom.fr (S.W.); fotios.stavrou@eurecom.fr (P.A.S.); 2 Division of Information Science and Engineering, KTH Royal Institute of Technology, 114 28 Stockholm, Sweden

**Keywords:** entropic optimal transport, Schrödinger problem, Talagrand inequality, entropy power inequality, log-concave measures

## Abstract

The distance that compares the difference between two probability distributions plays a fundamental role in statistics and machine learning. Optimal transport (OT) theory provides a theoretical framework to study such distances. Recent advances in OT theory include a generalization of classical OT with an extra entropic constraint or regularization, called entropic OT. Despite its convenience in computation, entropic OT still lacks sufficient theoretical support. In this paper, we show that the quadratic cost in entropic OT can be upper-bounded using entropy power inequality (EPI)-type bounds. First, we prove an HWI-type inequality by making use of the infinitesimal displacement convexity of the OT map. Second, we derive two Talagrand-type inequalities using the saturation of EPI that corresponds to a numerical term in our expressions. These two new inequalities are shown to generalize two previous results obtained by Bolley et al. and Bai et al. Using the new Talagrand-type inequalities, we also show that the geometry observed by Sinkhorn distance is smoothed in the sense of measure concentration. Finally, we corroborate our results with various simulation studies.

## 1. Introduction

OT theory studies how to transport one measure to another in the path with minimal cost. The Wasserstein distance is the cost given by the optimal path and closely connected with information measures; see, e.g., [1,2,3,4,5].

During the last decade, OT has been studied and applied extensively, especially in the machine learning community; see, e.g., [6,7,8,9]. Entropic OT, a technique to approximate the solution of the original OT, was given for computational efficiency in [10]. A key concept in the entropic OT is the Sinkhorn distance, which is a generalization of the Wasserstein distance with an extra entropic constraint. Due to the extra entropic constraint in the domain of the optimization problem, randomness is added to the original deterministic system, and the total cost increases from the original Wasserstein distance to a larger value. Therefore, a natural question is how to quantify the extra cost caused by the entropic constraint.

In this paper, we derive upper bounds for the quadratic cost of entropic OT, which are shown to include a term of entropy power responsible for quantifying the amount of uncertainty caused by the entropic constraint. This work is an extended version of [11].

### 1.1. Literature Review

The dynamical formulation of OT, also known as the Benamou–Brenier formula [12], generalizes the original Monge–Kantorovich formulation into a time-dependent problem. It changes the original distance problem (i.e., find the distance between two prescribed measures) into a geodesic problem (i.e., find the optimal path between two prescribed measures). Using the displacement convexity of relative entropy along the geodesic, functional inequalities such as HWI inequality and Talagrand inequality can be obtained (see, e.g., ([13] Chapter 20)).

Talagrand inequality, first given in [1], upper bounds the Wasserstein distance by relative entropy. Recent results in [2,4] obtain several refined Talagrand inequalities with dimensional improvements on the multidimensional Euclidean space. These inequalities bound Wasserstein distance with entropy power, which is sharper compared to the original one with relative entropy.

An analogue of the dynamical OT problem is the SP [14]. The SP aims to find the most likely evolution of a system of particles with respect to a reference process. The most likely evolution is called a Schrödinger bridge. SP and OT intersect on many occasions; see, e.g., [15,16,17]. The problem we study in this paper is in this intersection and mostly related to [15]. In particular, Léonard in [15] showed that the entropic OT with quadratic cost is equivalent to the SP with a Brownian motion as the reference process. He further derived that the Schrödinger bridge also admits a Benamou–Brenier formula with an additional diffusion term. Conforti in [18,19] claimed that the process can also be formulated as a continuity equation and proved that the acceleration of particles is the gradient of the Fisher information. The result therein leads to a generalized Talagrand inequality for relative entropy. Later, Bai et al. in [20] upper-bounded the extra cost from the Brownian motion by separating one Gaussian marginal into two independent random vectors. Using this approach, they showed that the dimensional improvement can be generalized to entropic OT and gave a Gaussian Talagrand inequality for the Sinkhorn distance. Additional results in [20] include a strong data processing inequality derived from their new Talagrand inequality and a bound on the capacity of the relay channel.

Entropic OT has other interesting properties. For example, Rigollet and Weed studied the case with one side of empirical measure in [21]. Their result shows that entropic OT performs maximum-likelihood estimation for Gaussian deconvolution of the empirical measure. This result can be further applied in uncoupled isotonic regression (see [9]). The dimensionality is also observed in the applications of entropic OT. For example, sample complexity bounds in [22,23] appear to be dimensional-dependent. In the GAN model, Reshetova et al. in [24] showed that the entropic regularization of OT promotes sparsity in the generated distribution.

Another element in our paper is EPI (for details on EPI, see, e.g., [25,26,27]). This inequality provides a clear expression to bound the differential entropy of two distributions’ convolution. We refer the interested reader to [28,29,30,31,32] for the connections between EPI and functional inequalities, and [33] for the connections between EPI and SP.

### 1.2. Contributions

**Figure 1:**
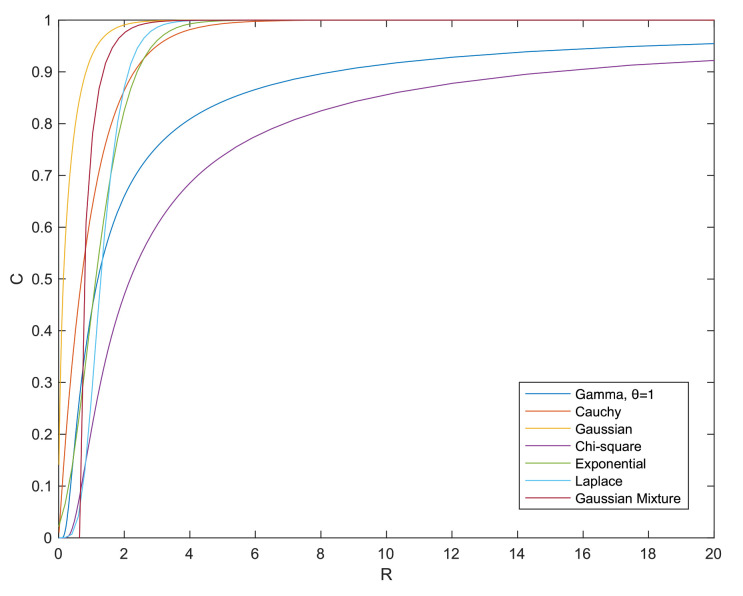
Plot of the numerical term *C* subject to the information constraint *R* evaluated with respect to different distributions for the one-dimensional case.

In this paper, we upper-bound the quadratic cost of entropic OT by deconvolution of one side measure and EPIs. Using this approach, we avoid any discussion related to the dynamics of SP and instead we capture the uncertainty caused by the Brownian motion quantitatively. Our contributions can be articulated as follows:

(1) We derive an HWI-type inequality for Sinkhorn distance using a modification of Bolley’s proof in [4] (see Theorem 2).
(2) We prove two new Talagrand-type inequalities (see Theorems 3 and 4). These inequalities are obtained via a numerical term *C* related to the saturation, or the tightness, of EPI. We claim that this term can be computed with arbitrary deconvolution of one side marginal, while the optimal deconvolution is shown to be unknown beyond the Gaussian case. Nevertheless, we simulate suboptimally this term for a variety of distributions in Figure 1.
(3) We show that the geometry observed by Sinkhorn distance is smoothed in the sense of measure concentration. In other words, Sinkhorn distance implies a dimensional measure concentration inequality following Marton’s method (see Corollary 2). This inequality has a simple form of normal concentration that is related to the term *C* and is weaker than the one implied by Wasserstein distance.
(4) Our theoretical results are validated via numerical simulations (see Section 4). These simulations reveal several reasons for which our bounds can be either tight or loose.

#### Connections to Prior Art

The novelty of our work is that it comprises naturally ideas from Bolley et al. in [4] and from Bai et al. in [20] to develop new entropic OT inequalities. The dimensional improvement of Bolley et al. in [4] separates an independent term of entropy power from the original Talagrand inequality. This allows us to utilize an approach to study the entropic OT problem, which is the OT with randomness, based on the convolutional property of entropy power. On the other hand, we generalize the constructive proof of Bai et al. in [20], where they separate one Gaussian random vector into two independent Gaussian random vectors. We further claim that, for any distribution, we can always find similar independent pairs satisfying several assumptions, to upper-bound the Sinkhorn distance. As a consequence of the above, our results generalize the Talagrand inequalities of Bolley et al. in ([4] Theorem 2.1) from classical OT to entropic OT and the results of Bai et al. in ([20] Theorem 2.2) from the Gaussian case to the strongly log-concave case. In particular, we show that Theorem 3 recovers ([4] Theorem 2.1) (see Corollary 1 and the discussion in Remark 6) and that Theorem 4 recovers ([20] Theorem 2.2) (see Remark 9). It should be noted that in our analysis, we focus on the primal problem defined in [10], as opposed to the studies of its Lagrangian dual in [18,19].

### 1.3. Notation

N is the set of positive integers {1,2,3,...}. R is the set of real numbers. $R^{n}$ is the *n*-dimension Euclidean space. $R_{+}$ denotes the set {x∈R:x≥0}.

Let X,Y be two Polish spaces, i.e., separable complete metric spaces. We write an element x∈X in lower-case letters and a random vector *X* on X in capital letters. We denote P(X) as the set of all probability measures on X. Let μ be a Borel measure on X. For a measurable map T:X→Y, $T_{\#}\mu$ denotes the pushing forward of μ to Y, i.e., for all A⊂Y, $T_{\#}\mu \left[A\right]=\mu \left[T^{-1}\left(A\right)\right]$. For p≥1, $L^{p}\left(X\right)$ or $L^{p}\left(d\mu \right)$ denotes the Lebesgue space of *p*-th order for the reference measure μ.

∇ is the gradient operator, ∇· is the divergence operator, Δ is the Laplacian operator, $D^{2}$ is the Hessian operator, $I_{n}$ is the *n*-dimension identity matrix, Id is the identity map, ∥·∥ is the Euclidean norm, $C^{k}$ is the set of functions that is *k*-times continuously differentiable, Ric is the Ricci curvature.

h(·), I(·;·), D(·∥·), J(·), I(·|·) denote differential entropy, mutual information, relative entropy, Fisher information and relative Fisher information, respectively. All the logarithms are natural logarithms. ∃! is unique existence. * is the convolution operator.

### 1.4. Organization of the Paper

The rest of the paper is organized as follows: in Section 2, we give the technical preliminaries of the theories and tools that we use; in Section 3, we state our main theoretical results; in Section 4, we give numerical simulations for our theorems, and in Section 5, we give the conclusions and future directions. Long proofs and background material are included in the Appendix.

## 2. Preliminaries

In this section, we give an overview of the theories and tools that we use.

### 2.1. Synopsis of Optimal Transport

We first give a brief introduction of OT theory. The OT problem was initialized by Gaspard Monge. The original formulation can be described as follows.

**Definition** **1** (Monge Problem [34]). *Let $P_{X}$ and $P_{Y}$ be two probability measures supported on two Polish spaces X, Y. Given a lower semi-continuous (see Definition A1) cost function $c\left(x,y\right):X\times Y\to R_{+}\cup \left\{+\infty \right\}$, the Monge problem wants to find a transport map T:X→Y minimizing the total cost:*

(1) $$\underset{T:T_{\#}P_{X}=P_{Y}}{\inf}\int_{X}c\left(x,T\left(x\right)\right)\;dP_{X}\left(x\right).$$

Then, Kantorovich gave a probabilistic interpretation to the OT. This is stated next.

**Definition** **2** (Kantorovich Problem [35]). *Let X and Y be two random vectors on two Polish spaces X, Y. X and Y have probability measures $P_{X}\in P\left(X\right)$, $P_{Y}\in P\left(Y\right)$. We denote $\Pi (P_{X},P_{Y})$ as the set of all joint probability measures on X×Y with marginal measures $P_{X}$, $P_{Y}$. Given a lower semi-continuous cost function $c\left(x,y\right):X\times Y\to R_{+}\cup \left\{+\infty \right\}$, the Kantorovich problem can be written as:*

(2) $$\underset{P\in \Pi (P_{X},P_{Y})}{\inf}\int_{X\times Y}c\left(x,y\right)\;dP.$$

It can be further proven that (2) gives the same optimizer as (1) (see, e.g., [36]). One can define the Wasserstein distance ([13] Definition 6.1) from (2). Let X=Y and let *d* be a metric on X. Then, the Wasserstein distance of order p,p≥1, is defined as follows:

(3) $$W_{p}\left(P_{X},P_{Y}\right):=\underset{P\in \Pi (P_{X},P_{Y})}{\inf}{\left[\int_{X\times Y}d^{p}\left(x,y\right)\;dP\right]}^{\frac{1}{p}}.$$

We note that the Wasserstein distance is a metric between two measures.

Cuturi in [10] gave the concept of entropic OT. In this definition, he adds an information theoretic constraint to (2), i.e.,

(4) $$\underset{P\in \Pi (P_{X},P_{Y};R)}{\inf}\int_{X\times Y}c\left(x,y\right)\;dP,$$

where

$$\Pi \left(P_{X},P_{Y};R\right):=\left\{P\in \Pi \left(P_{X},P_{Y}\right):I\left(X;Y\right)\leq R\right\},$$

with $I\left(X;Y\right):=D\left(P∥P_{X}\times P_{Y}\right)$ denoting the mutual information [32] between *X* and *Y*, and $R\in R_{+}$. It is well known that the constraint set is convex and compact with respect to the topology of weak convergence (for details, see, e.g., ([13] Lemma 4.4), ([37] Section 1.4)). Using the lower semi-continuity of c(x,y) and ([13] Lemma 4.3), we know that the objective function f:P→∫cdP is also lower semi-continuous. Using the compactness of the constraint set and the lower semi-continuity of *f*, then, from Weierstrass’ extreme value theorem, the minimum in (4) is attained. Moreover, the solution is always located on its boundary, i.e., I(X;Y)=R, because the objective function of (4) is linear.

Entropic OT is an efficient way to approximate solutions of the Kantorovich problem. The Lagrangian dual of (4), which was introduced by Cuturi in [10], can be solved iteratively. The dual problem of (4) can be reformulated as follows:

(5) $$\max_{\epsilon \geq 0}\underset{P\in \Pi (P_{X},P_{Y})}{\inf}\left\{\int_{X\times Y}c\left(x,y\right)\;dP+\epsilon \left(I(X;Y)-R\right)\right\},$$

where ϵ is a Lagrange multiplier. Using the Lagrange duality theorem ([38] Theorem 1, pp. 224–225), it can be shown that (4) and (5) give the same optimizer $P^{*}$.

The uncertainty of entropic OT can be understood as follows. We can write I(X;Y)=h(Y)−h(Y|X), where h(Y) is fixed. The conditional entropy encapsulates the randomness of the conditional distribution. The randomness decreases when I(X;Y) increases. Thus, unlike (1) and (2), there is no deterministic map anymore for (4) and (5), because a one-to-one mapping leads to infinite mutual information. Note that ϵ in (5) also has an explicit physical meaning. In particular, entropic OT with quadratic cost coincides with SP with a reference measure of Brownian motion (see [15]). Then, ϵ is a diffusion coefficient of the Fokker–Planck equation associated with the Schrödinger bridge.

In our main results, we study (4) instead of (5) for two reasons. First, the mutual information in (4) gives a global description of the amount of uncertainty, while the coefficient ϵ in (5) and its associated Fokker–Planck equation are more related to local properties, from the definitions of the Lagrangian dual and Fokker–Planck equation. Further on this point, there is no explicit expression for the correspondence between *R* and ϵ in the duality. Second, the expectation of cost function in (2) is comparable to the Wasserstein distance. As we demonstrate in the following, it gives a smooth version of the Wasserstein distance.

Similar to the Wasserstein distance, the Sinkhorn distance of order *p* is defined as follows:

(6) $$W_{p}\left(P_{X},P_{Y};R\right):=\underset{P\in \Pi (P_{X},P_{Y};R)}{\inf}{\left[\int_{X\times Y}d^{p}\left(x,y\right)\;dP\right]}^{\frac{1}{p}}.$$

Clearly, $\Pi (P_{X},P_{Y};R)$ is a subset of $\Pi (P_{X},P_{Y})$. Because of the minimization problem, it is easy to see that $W_{p}\left(P_{X},P_{Y};R\right)>W_{p}\left(P_{X},P_{Y}\right)$. For this reason, we say that entropic OT is a smoothed version of classical OT. We note that the Sinkhorn distance is not a metric because it does not fulfill the axiom of identity of indiscernibles.

Since entropic OT is concerned with mutual information, it may be of interest to introduce a conditional Sinkhorn distance. This is defined as follows:

(7) $$W_{p}\left(P_{X|Z},P_{Y|Z}|P_{Z};R\right):=\underset{P\in \Pi \left(P_{X|Z},P_{Y|Z}|P_{Z}\right);I\left(X;Y|Z\right)\leq R}{\inf}{\left\{E_{P}\left[d^{p}\left(X,Y\right)\right]\right\}}^{1/p},$$

where the conditional mutual information $I\left(X;Y|Z\right):=\int I\left(P_{X|Z=z};P_{Y|Z=z}\right)\;dP_{Z}\left(z\right)$ and $\Pi \left(P_{X|Z},P_{Y|Z}|P_{Z}\right):=\left\{P_{X,Y|Z}\cdot P_{Z}:P_{X,Y|Z=z}\in \Pi \left(P_{X|Z=z},P_{Y|Z=z}\right)\;\mathrm{for}\;z\;a.e.\right\}$. Conditional Sinkhorn distance is utilized in [20] and leads to a data processing inequality. Since the constraint of conditional mutual information is a linear form of $I(P_{X|Z=z};P_{Y|Z=z})$, the constraint set is still convex. The objective function is also a linear form of *P*. Therefore, the functional and topological properties of the conditional Sinkhorn distance are similar to the unconditional one.

Next, we state some known results of Talagrand inequality [1].

**Definition** **3** (Talagrand Inequality). *Let $P_{X}$ be a reference probability measure with density $e^{-V(x)}$, where V:X→R. We say that $P_{X}$ satisfies T(λ)>0, i.e., Talagrand inequality with parameter λ>0, if, for any $P_{Y}\in P\left(Y\right)$,*

(8) $$W_{2}\left(P_{X},P_{Y}\right)\leq \sqrt{\frac{2}{\lambda }D\left(P_{Y}∥P_{X}\right)}.$$

**Remark 1.** *(8) was originally introduced by Talagrand in [1] when $P_{X}$ is Gaussian. Blower in [39] gave a refinement and proved that*

$$D^{2}V\geq \lambda I_{n}\;\Rightarrow \;T\left(\lambda \right).$$

When going beyond the Euclidean space to a manifold, Otto and Villani in [40] showed that the Bakry–Emery condition $D^{2}V+\mathrm{Ric}$ also implies T(λ).

Recently, refined inequalities with dimensional improvements were obtained in multidimensional Euclidean space. These dimensional improvements were first observed in the Gaussian case of logarithmic Sobolev inequality, Brascamp–Lieb (or Poincaré) inequality [41] and Talagrand inequality [2]. For a standard Gaussian measure $P_{X}$, the dimensional Talagrand inequality has the form:

(9) $$W_{2}^{2}\left(P_{X},P_{Y}\right)\leq {E[∥Y∥}^{2}]+n-2ne^{\frac{1}{2n}{(E[∥Y∥}^{2}]-n-2D\left(P_{Y}∥P_{X}\right))}.$$

Bolley et al. in [4] generalized the results in [2,41] from Gaussian to strongly log-concave or log-concave. Next, we state their result. Let $dP_{X}=e^{-V}$, where $V:R^{n}\to R$ is $C^{2}$ continuous, $D^{2}V\geq \lambda I_{n}$. Bolley’s dimensional Talagrand inequality is given as follows:

(10) $$\begin{array}{cc} \frac{\lambda }{2}W_{2}^{2}\left(P_{X},P_{Y}\right) & \leq E\left[V\left(Y\right)\right]-E\left[V\left(X\right)\right]+n-ne^{\frac{1}{n}\left(E\left[V\left(Y\right)\right]-E\left[V\left(X\right)\right]-D\left(P_{Y}∥P_{X}\right)\right)}. \end{array}$$

The dimensional Talagrand inequalities (9) and (10) are tighter than (8). To see this result, one may refer to our Remark 6 below.

Bai et al. in [20] gave a generalization of (9) to Sinkhorn distance. When $P_{X}$ is standard Gaussian,

(11) $$W_{2}^{2}\left(P_{X},P_{Y};R\right)\leq {E[∥Y∥}^{2}]+n-2n\sqrt{\frac{1}{2\pi e}\left(1-e^{-\frac{2}{n}R}\right)}e^{\frac{1}{n}h\left(Y\right)}.$$

When R→+∞, this inequality coincides with (9).

### 2.2. Measure Concentration

The measure concentration phenomenon describes how the probability of a random variable *X* changes with the deviation from a given value such as its mean or median. Marton introduced an approach of concentration directly on the level of probability measures using OT (see, e.g., ([13] Chapter 22)).

To give the notation of concentration of measure, we first introduce the probability metric space. Let X be a Polish space. Let *d* be a metric on X. Let μ be a probability measure defined on the Borel set of X. Then, we say that the triple (X,d,μ) is a probability metric space.

For an arbitrary set A⊂X and any r≥0, we define $A_{r}$ as

$$A_{r}:=\left\{x\in X:d\left(x,A\right)>r\right\},$$

where $d\left(x,A\right):=\inf_{a\in A}d\left(x,a\right)$. Then, we say that a probability measure μ has normal (or Gaussian) concentration on (X,d) if there exists positive *K* and κ such that

(12) $$\mu \left(A\right)\geq \frac{1}{2}\;\Rightarrow \;\mu \left(A_{r}\right)\leq Ke^{-\kappa r^{2}},\;\forall r>0.$$

There is another weaker statement of normal concentration, such that

(13) $$\mu \left(A\right)\geq \frac{1}{2}\;\Rightarrow \;\mu \left(A_{r}\right)\leq Ke^{-\kappa {\left(r-r_{0}\right)}^{2}},\;\forall r>r_{0}.$$

It is not difficult to see that (12) can be obtained from (13), possibly with degraded constants, i.e., larger *K* and/or smaller κ.

The next theorem gives the connection between normal concentration and Talagrand inequality.

**Theorem** **1** (Theorem 3.4.7 [5]). *Let (X,d,μ) be a probability metric space. Then, the following two statements are equivalent:*

- *μ satisfies T(λ).*
- *μ has a dimension-free normal concentration with $\kappa =\frac{1}{2\lambda }$.*

The intuition behind Marton’s method is that OT theory can give a metric between two probability measures by the metric structure of the supporting Polish space. The metric can be further connected with probability divergence using Talagrand inequality.

### 2.3. Entropy Power Inequality and Deconvolution

EPI [25] states that, for all independent continuous random vectors *X* and *Y*,

(14) N(X+Y)≥N(X)+N(Y),

where $N\left(X\right):=\frac{1}{2\pi e}e^{\frac{2}{n}h\left(X\right)}$ denotes the entropy power of *X*. The equality is achieved when *X* and *Y* are Gaussian random vectors with proportional covariance matrices.

Deconvolution is a problem of estimating the distribution f(x) by the observations $Y_{1}$,...,$Y_{k}$ corrupted by additive noise $Z_{1}$,...,$Z_{k}$, written as

$$Y_{i}=X_{i}+Z_{i},$$

where k,i∈N and 1≤i≤k. $X_{i}$’s are i.i.d. in f(x), $Z_{i}$’s are i.i.d in h(z). $X_{i}$’s and $Z_{i}$’s are mutually independent. Let g(y) be the probability density function of *Y* that is given by the convolution g=f∗h. Then, their entropies can be bounded by EPI directly.

In our problem, we slightly abuse the concept by simply separating a random vector *Y* into two independent random vectors *X* and *Z*. We use this approach to introduce the uncertainty to entropic OT and consequently bound the Sinkhorn distance by EPI. Deconvolution is generally a more challenging problem than convolution. For instance, the log-concave family is convolution stable, i.e., convolution of two log-concave distributions is still log-concave, but we cannot guarantee that the deconvolution of two log-concave distributions is still log-concave. A trivial case is that wherein the deconvolution of a log-concave distribution by itself is a Dirac function. Moreover, *f* may not in general be positive or integrable for arbitrary given *g* and *h*, as shown in [42]. However, it should be noted that there are many numerical methods to compute deconvolution; see, e.g., [42,43,44].

## 3. Main Theoretical Results

In this section, we derive our main theoretical results. First, we give a new HWI-type inequality.

**Theorem** **2** (HWI-Type Inequality). *Let $X=Y=R^{n}$. Let μ be a probability measure with density $e^{-V(x)}$ with λ>0, where $V:R^{n}\to R$ is $C^{2}$ continuous, $D^{2}V\geq \lambda I_{n}$. Let $P_{X},P_{Y}$ be two probability measures on $R^{n}$, $P_{X},P_{Y}\ll \mu$. For any independent $Y_{1},Y_{2}$ satisfying $Y_{1}+Y_{2}=Y$, $E[Y_{2}]=0$ and $h\left(Y\right)-h\left(Y_{2}\right)\leq R$, the following bound holds:*

(15) $$\begin{array}{cc} \frac{\lambda }{2}W_{2}^{2}\left(P_{X},P_{Y};R\right) & \leq E\left[V\left(Y\right)\right]-E\left[V\left(X\right)\right]+n-n\;e^{\frac{1}{n}\left(h\left(Y_{1}\right)-h\left(X\right)\right)}+W_{2}\left(P_{X},P_{Y_{1}}\right)\sqrt{I(P_{X}|\mu )}, \end{array}$$

*where the relative Fisher information $I\left(P_{X}|\mu \right):=\int \frac{{∥\nabla f∥}^{2}}{f}d\mu ,f=\frac{dP_{X}}{d\mu }$*.

**Proof.**  See Appendix A. □

**Remark** **2.** *In Theorem 2, we construct $Y_{1}$ and $Y_{2}$, where $Y_{1}$ and X have a deterministic relationship. We note that the uncertainty in our construction, i.e., the independent $Y_{2}$, is located at one marginal, whereas the uncertainty of the true dynamics of the entropic OT is all along the path. The simplicity of our construction allows for the specific bound. We further note that there always exist such $Y_{1},Y_{2}$ satisfying the assumptions given in Theorem 2. A trivial proof is that $Y_{1}=E\left[Y\right]$ and $Y_{2}=Y-E\left[Y\right]$ fulfill the above assumptions.*

The next result gives a new Talagrand-type inequality.

**Theorem** **3** (Talagrand-Type Inequality). *Let $X=Y=R^{n}$. Let $dP_{X}=e^{-V(x)}dx$, where $V:R^{n}\to R$ is $C^{2}$ continuous, $D^{2}V\geq \lambda I_{n}$ with λ>0, $P_{Y}\ll P_{X}$. Then, the following bound holds:*

(16) $$\begin{array}{c} \frac{\lambda }{2}W_{2}^{2}\left(P_{X},P_{Y};R\right)\leq E\left[V\left(Y\right)\right]-E\left[V\left(X\right)\right]+n-nC\left(P_{Y},R\right)e^{\frac{1}{n}\left(h\left(Y\right)-h\left(X\right)\right)}, \end{array}$$

*where $C\left(P_{Y},R\right)\in \left[0,1\right]$ is a numerical term for the given $P_{Y}$ and R≥0.*

**Proof.**  Let $dP_{X}=e^{-V}$ in (15). In such a case, we have $I(P_{X}|\mu )=0$ from the definition of relative Fisher information. Take $C\left(P_{Y},R\right)=e^{\frac{1}{n}\left(h\left(Y_{1}\right)-h\left(Y\right)\right)}$; then, (16) is proven from (15). □

Next, we state some technical remarks on Theorem 3.

**Remark** **3** (On Theorem 3). *In Theorem 3, we show that the Sinkhorn distance of two random vectors can be upper-bounded by a difference of a functional on two marginals, i.e., E[V(Y)]−E[V(X)], and a term related to entropy power, i.e., $nC\left(P_{Y},R\right)e^{\frac{1}{n}\left(h\left(Y\right)-h\left(X\right)\right)}$. Interestingly, only the latter term is related to the constraint R. This means that the effect of information constraint is only associated with the randomness of the two random vectors instead of their positions. Recalling the physical meaning of entropic OT with quadratic cost, we can see that this expression is very natural, because the information constraint R is directly related to the randomness of the Schrödinger bridge.*

**Remark** **4** (On the numerical term C(·,·)). *The numerical term $C=e^{\frac{1}{n}\left(h\left(Y_{1}\right)-h\left(Y\right)\right)}$ can be computed by arbitrary $Y_{1}$ satisfying the assumptions that we gave in Theorem 2, i.e., $Y_{1},Y_{2}$ are independent, $Y_{1}+Y_{2}=Y$, $E[Y_{2}]=0$ and $h\left(Y\right)-h\left(Y_{2}\right)\leq R$. We observe that $e^{\frac{1}{n}h\left(Y_{1}\right)}$ has the form of a square root of entropy power. Using EPI and the fact that N(·)≥0, we have*

$$N\left(Y\right)\geq N\left(Y_{1}\right)+N\left(Y_{2}\right)\geq N\left(Y_{1}\right).$$

*Therefore, $C=e^{\frac{1}{n}\left(h\left(Y_{1}\right)-h\left(Y\right)\right)}=\sqrt{N\left(Y_{1}\right)/N\left(Y\right)}\in \left[0,1\right]$. When R=0, then $Y_{2}=Y-E\left[Y\right]$ and the density of $Y_{1}$ is δ(x−E[Y]). This means that $e^{\frac{1}{n}h\left(Y_{1}\right)}=0$, hence C=0. When R=+∞, then $Y=Y_{1}$, $e^{\frac{1}{n}h\left(Y_{1}\right)}=e^{\frac{1}{n}h\left(Y\right)}$, and consequently C=1. Therefore, C(·,0)=0, C(·,+∞)=1 for all $P_{Y}$.*

*Moreover, we can show that there always exists such a sequence C non-decreasing with respect to R. We know that $C=e^{\frac{h(Y_{1})}{n}-\frac{h(Y)}{n}}$ subject to $Y_{1}+Y_{2}=Y$, $E[Y_{2}]=0$ and $h\left(Y\right)-h\left(Y_{2}\right)\leq R$. Thus, for a larger value of R, there exists at least a $h(Y_{2})$ non-increasing. This further leads to a non-decreasing $h(Y_{1})$. Therefore, there exists at least a C(·,R+ΔR) not smaller than C(·,R), ∀ΔR>0, i.e., C(·,R) is monotonic non-decreasing with respect to R.*

*We note that, for particular distributions, we may have an explicit expression of C(·,·). For instance, when $P_{Y}$ is Gaussian, we can always take the linear combination $Y=Y_{1}+Y_{2}$, where $Y_{1}$ and $Y_{2}$ are independent Gaussian and have proportional covariance matrices. In such a case, EPI is saturated as follows:*

$$\begin{array}{cc} e^{\frac{2}{n}h\left(Y_{1}\right)} & =e^{\frac{2}{n}h\left(Y\right)}-e^{\frac{2}{n}h\left(Y_{2}\right)}=\left(1-e^{-\frac{2}{n}R}\right)e^{\frac{2}{n}h\left(Y\right)}. \end{array}$$

*As a result, we have $C\left(P_{Y},R\right)=e^{\frac{1}{n}\left(h\left(Y_{1}\right)-h\left(Y\right)\right)}=\sqrt{1-R^{\frac{2}{n}}}$. For Cauchy distribution $Cauchy(x_{0},\gamma )$, its differential entropy is log(4πγ). The summation of independent Cauchy random variables $\sum_{i}^{n}Cauchy\left(x_{i},\gamma_{i}\right)∼Cauchy\left(\sum_{i}^{n}x_{i},\sum_{i}^{n}\gamma_{i}\right)$. When Y is i.i.d. Cauchy, i.e., ${\left(Y\right)}_{i}∼Cauchy\left(x_{0},\gamma \right)$, we take ${\left(Y_{1}\right)}_{i}∼Cauchy\left(x_{0},\frac{1}{4\pi }e^{\frac{1}{n}h\left(Y\right)}\cdot \left(1-e^{-\frac{R}{n}}\right)\right)$ and ${\left(Y_{2}\right)}_{i}∼Cauchy\left(0,\frac{1}{4\pi }e^{\frac{1}{n}h\left(Y\right)}\cdot e^{-\frac{R}{n}}\right)$. We can see that this linear combination satisfies our assumption $h\left(Y\right)-h\left(Y_{2}\right)\leq R$ and $C\left(P_{Y},R\right)=e^{\frac{1}{n}\left(h\left(Y_{1}\right)-h\left(Y\right)\right)}=1-e^{-\frac{R}{n}}$.*

*Note that the linear combination $Y=Y_{1}+Y_{2}$ is not unique, according to the assumption of Theorem 2. Consequently, this implies the non-uniqueness of C(·,·). In order to obtain the tightest bound in (16), we need to solve the following optimization problem*

(17) $$C^{*}\left(P_{Y},R\right)=\sup e^{\frac{1}{n}\left(h\left(Y_{1}\right)-h\left(Y\right)\right)},$$

*subject to $Y_{1}+Y_{2}=Y$ and $h\left(Y\right)-h\left(Y_{2}\right)\leq R$. To look into this optimization problem, we recall Courtade’s reverse EPI ([31] Corollary 1) as follows. If we have independent X and Y with finite second moments and choose θ to satisfy θ/(1−θ)=N(Y)/N(X), then*

(18) N(X+Y)≤(N(X)+N(Y))(θp(X)+(1−θ)p(Y)),

*where $p\left(X\right):=\frac{1}{n}N\left(X\right)J\left(X\right)\geq 1$ is the Stam defect and $J\left(X\right):=I\left(P_{X}|\mu \right)$, where dμ=dx is the Fisher information. We note that p(X) is affine invariant, i.e., p(X)=p(tX), t>0 because $t^{2}N\left(X\right)=N\left(tX\right)$ and $t^{2}J\left(tX\right)=J\left(X\right)$. We note that the equality p(X)=1 holds only if X is Gaussian. In our case, $\theta =N\left(Y_{1}\right)/\left(N\left(Y_{1}\right)+N\left(Y_{2}\right)\right)$. When θ→1, (18) becomes*

$$N\left(Y\right)≲\left(N\left(Y_{1}\right)+N\left(Y_{2}\right)\right)\cdot p\left(Y_{2}\right).$$

*This means that the saturation of EPI is controlled by $p(Y_{2})$ when the noise $Y_{2}$ is small, i.e., when R is large. In such a case, $C^{*}\left(P_{Y},R\right)\approx \sqrt{1-R^{\frac{2}{n}}}$ if we let $Y_{2}$ be close to Gaussian, i.e., $p(Y_{2})=1$. On the other hand, when θ→0, EPI can also be saturated if we let $Y_{1}$ be close to Gaussian.*

*In Figure 1, we illustrate numerical simulations of C(·,·) for the one-dimensional case. For general distributions beyond Gaussian and i.i.d. Cauchy, one can approximate C(·,·) using kernel methods of deconvolution; see, e.g., [43,44]. Our strategy of deconvolution in Figure 1 is to let $Y_{2}=tY^{\prime }$, where $Y^{\prime }$ is a copy of Y and t∈[0,1]. Gaussian mixture is an exception for this strategy because its spectrum would not be integrable. Instead, we let $Y_{2}$ be Gaussian for a Gaussian mixture. We note that this strategy is mostly not optimal and the optimal way to maximize the entropy power in (17) remains an open question.*

**Remark** **5** (On the condition of identity of Theorem 3). *To show the condition of identity of (16), we need the inequalities in (A1) and in Lemma A1 to be equalities. The equality of (A1) holds when $P_{X}$ is isotropic Gaussian, i.e., $P_{X}∼N\left(\mu ,\sigma^{2}I_{n}\right)$ for some $\mu \in R^{n}$ and σ>0. The equality in Lemma A1 holds when ∇φ is affine and $D^{2}\varphi$ has identical eigenvalues, i.e., ∇φ=k·Id,k∈R, see ([4] Lemma 2.6). From ([45] Theorem 1), we know that the linear combination $Y=Y_{1}+Y_{2}$ in Theorem 2 is the optimizer for entropic OT when X and Y are isotropic Gaussian. In such a case, the equality of (16) holds and $C\left(\cdot ,R\right)=\sqrt{1-R^{\frac{2}{n}}}$.*

The following corollary is immediate from Theorem 3.

**Corollary** **1.** *Wasserstein distance is bounded by*

(19) $$\frac{\lambda }{2}W_{2}^{2}\left(P_{X},P_{Y}\right)\leq E\left[V\left(Y\right)\right]-E\left[V\left(X\right)\right]+n-ne^{\frac{1}{n}\left(h\left(Y\right)-h\left(X\right)\right)}.$$

**Proof.**  This is immediate from Theorem 3 when R→∞. In this case, C(·,+∞)=1. □

**Remark** **6** (On Corollary 1). *We note that (19) is equivalent to Bolley’s dimensional Talagrand inequality (10) and it is tighter than the classical Talagrand inequality (8). To make this point clear, note that, under our assumptions, h(X)=E[V(X)] and $D\left(P_{Y}∥P_{X}\right)=E\left[V\left(Y\right)\right]-h\left(Y\right)$ because $dP_{X}=e^{-V(x)}dx$. Clearly, by substituting these expressions to the last term of (19), we obtain (10). Since $e^{\mu }\geq 1+\mu$, (10) is, in general, tighter than the classical Talagrand inequality (8), i.e., RHS of (10) ≤ RHS of (8). The equality holds if and only if h(Y)=h(X).*

We notice that *C* is the only difference between (10) and (16), from Remark 6. Therefore, we can immediately obtain a result related to measure concentration following ([4] Corollary 2.4). Next, we state the result on measure concentration obtained from (16).

**Corollary** **2.** *Let $d\mu =e^{-V}$, where $V:R^{n}\to R$ is $C^{2}$ continuous, $D^{2}V\geq \lambda I_{n}$ with λ>0. Let $A\subset R^{n}$, $A_{r}:=\{x\in R^{n}:\forall y\in A,∥x-y∥>r\}$ for r≥0 and $c_{A}:=\sqrt{2\lambda^{-1}\log\left(1/\mu \left(A\right)\right)}$. Then, for $r\geq c_{A}$, we obtain*

(20) $$\mu \left(A_{r}\right)\leq C^{-n}\cdot e^{-\frac{\lambda }{2}{\left(r-c_{A}\right)}^{2}}.$$

**Proof.**  See Appendix B. □

Next, we state some technical comments on Corollary 2.

**Remark** **7** (On Corollary 2). *We note that in the derivation of Corollary 2, we follow the method of Marton in [46], which utilizes the geometrical properties of Wasserstein distance. From our discussion above, the information constraint leads to the uncertainty in entropic OT. In this result, we further show that the uncertainty smooths the geometrical properties of Wasserstein distance, i.e., Sinkhorn distance implies a looser measure concentration inequality. We begin with two extreme cases. When C=0, the two random vectors are independent and entropic OT has the most uncertainty. It is natural that the quadratic difference of two independent random vectors does not imply any concentration. When C=1, the inequality is the same as the one in Theorem 1. Between these two extremes, i.e., when 0<C<1, Sinkhorn distance leads to a weaker normal concentration, compared to Theorem 1. Furthermore, we include in Appendix C the proof that demonstrates that the Sinkhorn distance gives a weaker measure concentration inequality in high dimensions.*

The next theorem is another Talagrand-type inequality. Compared to Theorem 3, the following result is a bound obtained using a term related to the saturation of $P_{X}$, instead of the saturation of $P_{Y}$ that was used in Theorem 3.

**Theorem** **4.** *Let $X=Y=R^{n}$. Without loss of generality, let X be a zero-mean random vector with density $e^{-V(x)}$, where $V:R^{n}\to R$ is $C^{2}$ continuous, $D^{2}V\geq \lambda I_{n}$ with λ>0, $P_{Y}\ll P_{X}$. Then, the following bound holds:*

(21) $$\begin{array}{c} \frac{\lambda }{2}W_{2}^{2}\left(P_{X},P_{Y};R\right)\leq E\left[V\left(Y\right)\right]-E\left[V\left(X\right)\right]+n-nC_{x}\left(P_{X},R\right)e^{\frac{1}{n}\left(h\left(Y\right)-h\left(X\right)\right)}+\epsilon^{\prime }, \end{array}$$

*where $\epsilon^{\prime }$ is a term related to the linearity of V.*

**Proof.**  See Appendix D. □

We offer the following technical comments on Theorem 4.

**Remark** **8** (On Theorem 4). *Similar to $C(P_{Y},R)$, $C_{x}\left(P_{X},R\right)\in \left[0,1\right]$ can be computed by the equation $X=C_{x}X^{\prime }+X_{2}$ with arbitrary independent $X^{\prime },X_{2}$ under the assumptions that $X^{\prime }$ is a copy of X, $E[X_{2}]=0$, $h\left(X\right)-h\left(X_{2}\right)\leq R$, as shown in the proof. However, (21) is less natural than (16) because of the extra term $\epsilon^{\prime }$. When ∇V is nearly linear, $\epsilon^{\prime }$ should be small. When ∇V is far from linear, $\epsilon^{\prime }$ is unknown.*

*Theorem 4 can also give a measure concentration inequality, namely*

(22) $$\mu \left(A_{r}\right)\leq C_{x}^{-n}\cdot e^{-\frac{\lambda }{2}{\left(r-c_{A}\right)}^{2}+\epsilon^{\prime }},$$

*where $A_{r}$ and $c_{A}$ are the same as those defined in Corollary 2. We omit the proof of (22) because it follows using similar steps to the ones used to prove Corollary 2.*

**Remark** **9.** *When ∇V is linear, $\epsilon^{\prime }$ is zero and $C_{x}\left(\cdot ,R\right)=\sqrt{1-R^{\frac{2}{n}}}$, as simply taking $t=\sqrt{1-R^{\frac{2}{n}}}$ in the proof. In such a case, (21) recovers (11) by taking X as a standard Gaussian, i.e., $V\left(x\right)={∥x∥}^{2}/2+k$, where k is a normalization factor. Substitute V and times 2 on both sides of (21), we have*

(23) $$\begin{array}{cc} W_{2}^{2}\left(X,Y;R\right)\leq & {E[∥Y∥}^{2}{]-E[∥X∥}^{2}]+2n-2n\sqrt{1-e^{-\frac{2}{n}R}}e^{\frac{1}{n}\left(h\left(Y\right)-h\left(X\right)\right)}\\ = & {E[∥Y∥}^{2}]+n-2n\sqrt{\frac{1}{2\pi e}\left(1-e^{-\frac{2}{n}R}\right)}e^{\frac{1}{n}h\left(Y\right)}, \end{array}$$

*which is exactly the same as (11).*

The next theorem gives a Talagrand-type bound for the conditional Sinkhorn distance.

**Theorem** **5** (Talagrand-type bound for conditional Sinkhorn distance). *Let $X=Y=R^{n}$. Given a probability measure $P_{Z}$ and two conditional probability measures $P_{X|Z}$ and $P_{Y|Z}$, where the probability density $dP_{X|Z=z_{0}}=dP_{X}=e^{-V(x)}dx,\forall z_{0}\in Z$, let $V:R^{n}\to R$ be $C^{2}$ continuous, $D^{2}V\geq \lambda I_{n}$ with λ>0, $P_{Y|Z=z_{0}}\ll P_{X}$. Then, the following bound holds:*

(24) $$\begin{array}{c} \frac{\lambda }{2}W_{2}^{2}\left(P_{X},P_{Y}|P_{Z};R\right)\leq E\left[V\left(Y|Z\right)\right]-E\left[V\left(X\right)\right]+n-nC^{\prime }\left(P_{Y|Z},R\right)e^{\frac{1}{n}\left(h\left(Y|Z\right)-h\left(X\right)\right)}, \end{array}$$

*where $C^{\prime }\left(P_{Y|Z},R\right)$ is a numerical term. A direct observation is that $C^{\prime }\left(P_{Y|Z},R\right)$ can take $\inf_{z_{0}\in Z}C\left(P_{Y|Z=z_{0}},R\right)$.*

**Proof.**  See Appendix E. □

## 4. Numerical Simulations

**Figure 2:**
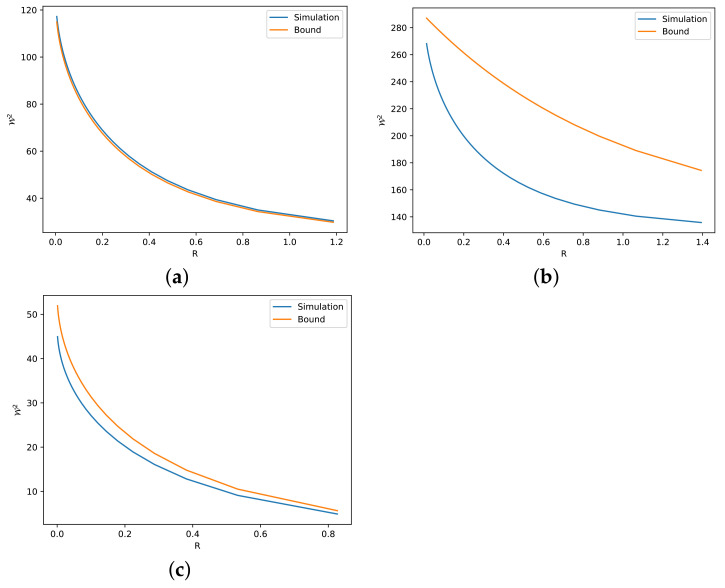
Numerical simulations and bounds via (16) for different *R*. (**a**) $dP_{X}∼N\left(0,\frac{1}{25}\right)$ and $dP_{Y}∼N\left(0,\frac{1}{100}\right)$. (**b**) $dP_{X}∼N\left(0,\frac{1}{25}\right)$ and $dP_{Y}∼Cauchy\left(0,10\right)$. (**c**) $dP_{X}=e^{-V},V={\left(x/5\right)}^{2}/2+\left|x/10\right|+e^{-|x/10|}+k,k\in R$ and $dP_{Y}∼N\left(0,\frac{1}{25}\right)$.

In this section, we describe several numerical simulations to illustrate the validity of our theoretical findings. To check the tightness of our bounds, we use as a reference bound the numerical solution obtained via the Sinkhorn algorithm, which can be found in the POT library [47]. As an iterative method, the Sinkhorn algorithm has computational error, since the iteration stops when it converges to a certain rate. For example, in Figure 2a, we plot the result for Theorem 3 with two Gaussian marginals, which is the scenario when the identity of (16) holds. From the figure, we can see that the simulation is slightly greater than the bound. Nevertheless, we note that the precision is reasonably small.

**Figure 3:**
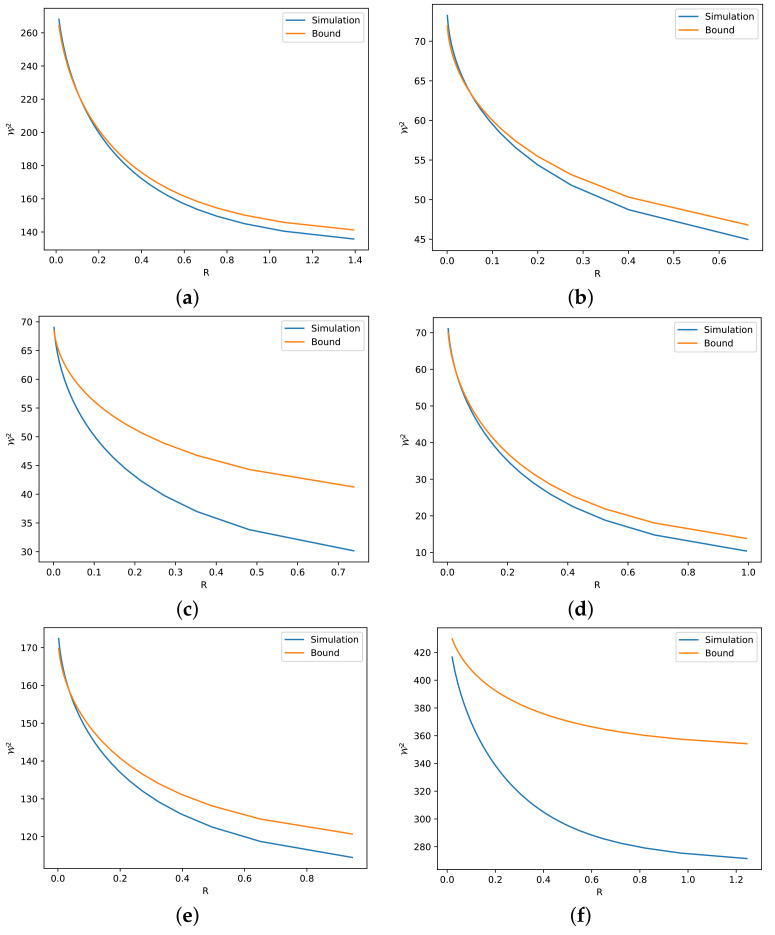
Numerical simulations and bounds via (21) for different *R*. (**a**) $dP_{X}∼N\left(0,\frac{1}{25}\right)$ and $dP_{Y}∼Cauchy\left(0,10\right)$. (**b**) $dP_{X}∼N\left(0,\frac{1}{25}\right)$ and $dP_{Y}∼\chi^{2}\left(6\right)$. (**c**) $dP_{X}∼N\left(0,\frac{1}{25}\right)$ and $dP_{Y}∼Exp\left(0.2\right)$. (**d**) $dP_{X}∼N\left(0,\frac{1}{25}\right)$ and $dP_{Y}∼Laplace\left(0,5\right)$. (**e**) $dP_{X}∼N\left(0,\frac{1}{25}\right)$ and $dP_{Y}$ is Gamma distribution with α=2 and β=0.2. (**f**) $dP_{X}∼N\left(0,\frac{1}{25}\right)$ and $dP_{Y}=\frac{1}{2}N\left(-20,\frac{1}{25}\right)+\frac{1}{2}N\left(20,\frac{1}{25}\right)$.

The simulations for Theorems 3 and 4 are given in Figure 2 and Figure 3, respectively. Since the optimal value of *C* in Theorem 3 and the error term $\epsilon^{\prime }$ in Theorem 4 beyond the linear case are unknown, we mainly simulate the case with one side Gaussian, i.e., with $C=\sqrt{1-R^{\frac{2}{n}}}$ and $\epsilon^{\prime }=0$. In this way, we avoid the unknown factors and deduce several observations related to the tightness of the bounds derived in these two theorems.

**Figure 4:**
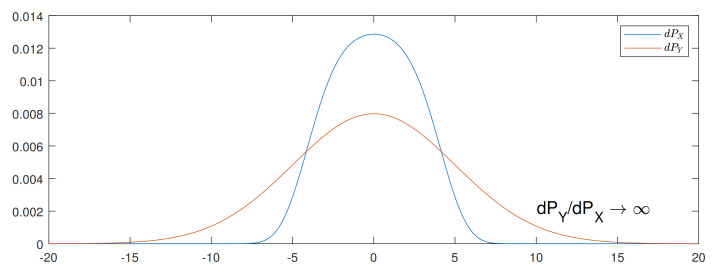
Probability densities of $dP_{X}=e^{-V},V={\left(x/5\right)}^{2}/2+{\left(x/5\right)}^{4}+k,k\in R$ and $dP_{Y}∼N\left(0,\frac{1}{25}\right)$.

**Figure 5:**
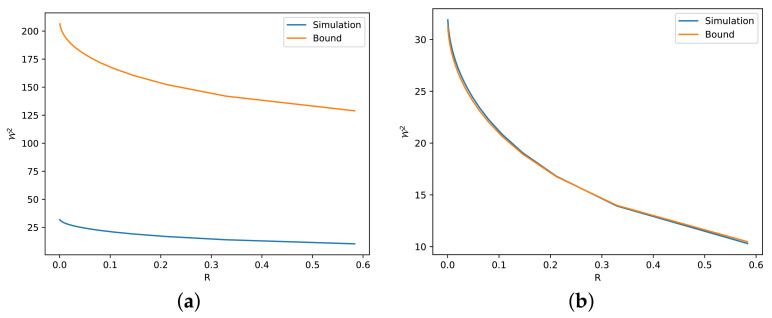
Numerical simulations and bounds for different *R*, with $d\mu =e^{-V},V={\left(x/5\right)}^{2}/2+{\left(x/5\right)}^{4}+k,k\in R$ and $d\mu ∼N(0,\frac{1}{25})$. (**a**) Bound via (16) with $dP_{X}=d\mu$, $dP_{Y}=d\nu$. (**b**) Bound via (21) with $dP_{X}=d\nu$, $dP_{Y}=d\mu$.

**Figure 6:**
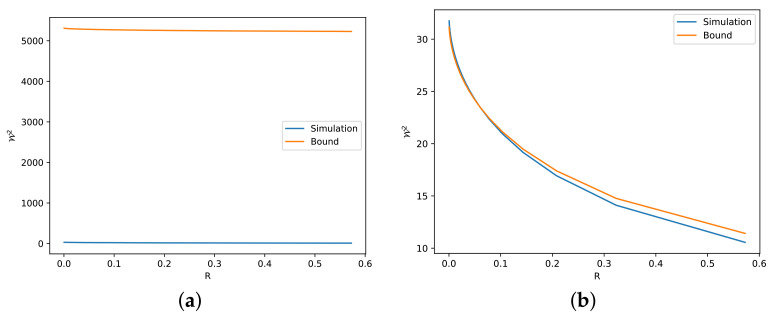
Numerical simulations and bounds for different *R*, with $d\mu =e^{-V},V={\left(x/5\right)}^{2}/2+{\left(x/5\right)}^{8}+k,k\in R$ and $d\mu ∼N(0,\frac{1}{25})$. (**a**) Bound via (16) with $dP_{X}=d\mu$, $dP_{Y}=d\nu$. (**b**) Bound via (21) with $dP_{X}=d\nu$, $dP_{Y}=d\mu$.

The first observation is about absolute continuity. We observe that the original Talagrand inequality (8) is not tight when $P_{Y}$ is not absolutely continuous with respect to $P_{X}$, because $D(P_{Y}∥P_{X})=+\infty$ in this case. In Figure 4, we illustrate one such case with almost discontinuity between two strongly log-concave distributions, i.e., the Radon–Nikodym derivative $dP_{Y}/dP_{X}=\exp\left[{\left(x/5\right)}^{4}\right]+k^{\prime }$, where $k^{\prime }\in R$ is a normalizing factor, goes to *∞* when x→∞. Consequently, the bound (16) from Theorem 3 is loose, as illustrated in Figure 5. The bound can be much looser if we increase the discontinuity, i.e., we let $dP_{Y}/dP_{X}=\exp\left[{\left(x/5\right)}^{8}\right]+k^{\prime }$, as shown in Figure 6. By simply changing the sides of distributions $P_{X}$ and $P_{Y}$, we preserve the absolute continuity and the bound becomes tight, as we can see in Figure 5b and Figure 6b.

The second observation is related to the numerical term *C*. By comparing Figure 2b and Figure 3a, we observe that $C=\sqrt{1-R^{\frac{2}{n}}}$ gives a better description than $C=1-R^{\frac{1}{n}}$, i.e., the former one gives a tighter bound. This is reasonable according to our previous discussion, i.e., the independent linear combination of Cauchy random variables is not the optimal deconvolution. Actually, even if $P_{Y}$ is not Gaussian in (16), $C=\sqrt{1-R^{\frac{2}{n}}}$ seems to be true for all the simulated distributions.

Furthermore, we observe that the tightness of the bounds in Theorems 3 and 4 is related to the linearity of the transport map, which can be seen as a similarity between the two marginals. For example, Cauchy and Laplace distributions are similar to the Gaussian distribution. Thus, they show a tight bound in Figure 3a,d. On the other hand, Gaussian mixture and exponential distribution are relatively far from the Gaussian distribution. Hence, Figure 3c,f give looser bounds.

**Figure 7:**
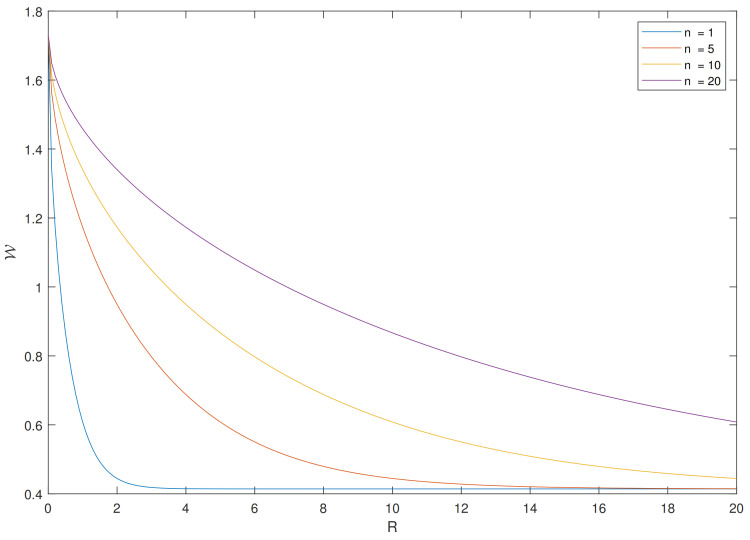
Sinkhorn distances between isotropic Gaussians in different dimensions.

In Figure 7, we plot the dimensionality of Sinkhorn distance between isotropic Gaussians. Different curves correspond to a pair of Gaussian distributions in different dimensions and these pairs have the same Wasserstein distance. It can be seen that the information constraint causes more smoothing in higher dimensions, which is consistent with Corollary 2.

## 5. Conclusions and Future Directions

In this paper, we considered a generalization of OT with an entropic constraint. We showed that the constraint leads to uncertainty and the uncertainty can be captured by EPI. We first derived an HWI-type inequality for the Sinkhorn distance. Then, we derived two Talagrand-type inequalities. Because of the strong geometric implication of Talagrand inequality, these two Talagrand-type inequalities can also give a weaker measure concentration inequality, respectively. From this result, we claimed that the geometry implied by the Sinkhorn distance can be smoothed out by the entropic constraint. We also showed that our results can be generalized into a conditional version of entropic OT inequality.

However, there are two factors unknown in the inequalities we derived, i.e., the optimal value of the term *C* in Theorem 3 and the error term $\epsilon^{\prime }$ in Theorem 4 when one goes beyond the linear case. Although we showed that we can compute a suboptimal *C* using the arbitrary linear combination of two random vectors, the optimal value $C^{*}$ is an intriguing open question to answer. We believe that the improvement of the term *C* may be related to the Fisher information. Without the assumption of strong log-concavity, it requires an extra term of relative Fisher information to upper-bound the Wasserstein distance in Theorem 2. The reversing of EPI in [31] is also concerned with Fisher information. If we consider the changing of Fisher information along the Schrödinger bridge, a better estimate of term *C* may be feasible.

## Data Availability

Not applicable.

## Abbreviations

The following abbreviations are used in this manuscript:

OT	optimal transport
EPI	entropy power inequality
SP	Schrödinger problem
GAN	generative adversarial network
RHS	right-hand side
a.e.	almost everywhere
POT	Python Optimal Transport

## Appendix A. Proof of Theorem 2

For a $C^{2}$ continuous function $V:R^{n}\to R$, $D^{2}V\geq \lambda I_{n}$, by Taylor formula ([4] Lemma 2.5), there exists a t∈[0,1] satisfying

(A1) $$\begin{array}{cc} V(y)-V(x)= & \nabla V\left(x\right)\cdot \left(y-x\right)+\left(y-x\right)\cdot D^{2}V\left(tx+\left(1-t\right)y\right)\left(y-x\right)/2\\ \geq & \nabla V\left(x\right)\cdot \left(y-x\right)+\frac{\lambda }{2}{∥y-x∥}^{2}. \end{array}$$

Hence, we can bound the second-order cost by

(A2) $$\frac{\lambda }{2}\int_{X\times Y}{∥y-x∥}^{2}\;dP\leq \int_{X\times Y}V\left(y\right)-V\left(x\right)-\nabla V\left(x\right)\cdot \left(y-x\right)\;dP.$$

Because entropic OT is a minimization problem, we can take any case in $\Pi (P_{X},P_{Y};R)$ to bound $W_{2}\left(P_{X},P_{Y};R\right)$. We take a linear combination $Y=Y_{1}+Y_{2}$, where $Y_{1}$ and $Y_{2}$ are independent, $h\left(Y\right)-h\left(Y_{2}\right)\leq R$ and $E[Y_{2}]=0$. Assume that there is a Brenier map between $Y_{1}$ and *X*, i.e., $Y_{1}=\nabla \varphi \left(X\right)$, which always exists, according to Theorem A1 (see Appendix F). Then, we can see that this special case is in $\Pi (P_{X},P_{Y},R)$, namely,

$$\begin{array}{cc} I(X;Y) & =h(Y)-h(Y|X)\\ \; & =h\left(Y\right)-h\left(Y_{1}+Y_{2}|X\right)\\ \; & =h\left(Y\right)-h\left(Y_{2}\right)\\ \; & \leq R. \end{array}$$

Let $d\mu =e^{-V}$, where $V:R^{n}\to R$ is $C^{2}$ continuous, $D^{2}V\geq \lambda I_{n}$. In order to bound the Sinkhorn distance, we simply need to bound $\int_{X\times Y}\nabla V\left(x\right)\cdot \left(y-x\right)\;dP$, according to (A2). This term can be bounded as follows:

(A3) $$\begin{array}{cc} \int_{X\times Y}\nabla V\left(x\right)\cdot \left(y-x\right)\;dP= & \;\int \int \;\nabla V\left(x\right)\cdot \left(\nabla \varphi \left(x\right)+y_{2}-x\right)\;dP_{Y_{2}}\;dP_{X}\\ = & \int \nabla V\left(x\right)\cdot \left(\nabla \varphi \left(x\right)-x\right)\;dP_{X}\\ = & \int \nabla V\left(x\right)\cdot \left(\nabla \varphi \left(x\right)-x\right)\frac{dP_{X}}{d\mu }\;d\mu \\ \geq & \int \Delta \varphi (x)f\;d\mu -n+\int (\nabla \varphi (x)-x)\cdot \nabla f\;d\mu \\ \geq & ne^{\frac{1}{n}\left(h\left(Y_{1}\right)-h\left(X\right)\right)}-n-W_{2}\left(P_{X},P_{Y_{1}}\right)\cdot \sqrt{I(P_{X}|\mu )}, \end{array}$$

where we take the Radon–Nikodym derivative $f=\frac{dP_{X}}{d\mu }$ in (A3) and apply Lemma A1 in Appendix F. This completes the derivation.

## Appendix B. Proof of Corollary 2

Let $\mu_{A}=\frac{1_{A}}{\mu (A)}\mu$ and $\mu_{A_{r}}=\frac{1_{A_{r}}}{\mu (A_{r})}\mu$ be the conditional probability measure μ restricted to *A* and $A_{r}$. Using the triangle inequality of $W_{2}$, we have

(A4) $$r\leq W_{2}\left(\mu_{A},\mu_{A_{r}};R\right)\leq W_{2}\left(\mu_{A},\mu \right)+W_{2}\left(\mu ,\mu_{A_{r}};R\right).$$

From (8), we know that

$$W_{2}\left(\mu_{A},\mu \right)\leq \sqrt{2\lambda^{-1}D\left(\mu_{A}∥\mu \right)}=\sqrt{2\lambda^{-1}\log\left(1/\mu \left(A\right)\right)}:=c_{A}.$$

Let μ and $\mu_{A}$ be the probability measures of two random vectors *X* and *Y*, respectively. By substituting $c_{A}$ into (A4) and using (16), we have

$$\begin{array}{cc} {\left(r-c_{A}\right)}^{2}\leq & W_{2}^{2}\left(\mu ,\mu_{A_{r}};R\right)\\ \leq & \frac{2}{\lambda }\left(E\left[V\left(Y\right)\right]-E\left[V\left(X\right)\right]+n-nCe^{\frac{1}{n}\left(h\left(Y\right)-h\left(X\right)\right)}\right)\\ = & \frac{2}{\lambda }\left(E\left[V\left(Y\right)\right]-E\left[V\left(X\right)\right]+n-nCe^{\frac{1}{n}\left(E\left[V\left(Y\right)\right]-E\left[V\left(X\right)\right]-D\left(\mu_{A_{r}}∥\mu \right)\right)}\right). \end{array}$$

Substituting $\mu_{A_{r}}=\frac{1_{A_{r}}}{\mu (A_{r})}\mu$ into the definition of relative entropy, we have

$$\begin{array}{cc} D(\mu_{A_{r}}∥\mu )= & \int_{R^{n}}\log\frac{1_{A_{r}}}{\mu (A_{r})}\cdot \frac{1_{A_{r}}}{\mu (A_{r})}\;d\mu \\ = & -\frac{\log\mu (A_{r})}{\mu (A_{r})}\int_{A_{r}}d\mu \\ = & -\log\mu (A_{r}). \end{array}$$

Then, we obtain, for $r\geq c_{A}$,

(A5) $$\mu \left(A_{r}\right)\leq C^{-n}\cdot e^{E[V(X)]-E[V(Y)]}{\left[1+\frac{1}{n}\left(E\left[V\left(Y\right)\right]-E\left[V\left(X\right)\right]-\frac{\lambda }{2}{\left(r-c_{A}\right)}^{2}\right)\right]}^{n}.$$

(A5) already indicates a concentration of measure. Using the inequality ${\left(1+u/n\right)}^{n}\leq e^{u}$, it can be further shown that (A5) also implies normal concentration, as follows:

$$\begin{array}{cc} \; & \mu \left(A_{r}\right)\leq C^{-n}\cdot e^{-\frac{\lambda }{2}{\left(r-c_{A}\right)}^{2}}. \end{array}$$

## Appendix C. Proof of the Dimensionality of (20)

To prove that (20) is weaker when the dimension increases, we only need to show the decreasing of $C^{n}$ with respect to *n*. When we increase the dimension, for given random vectors $Y_{1}+Y_{2}=Y$, we assume that this convolution relation still holds and their entropies increase proportionally to *n*, i.e., the shapes of their distributions do not change.

Since now *n* is a variable of *C*, we let n∈R and let *C* be a function with respect to $\frac{R}{n}$, in order to compute the partial derivative. We first show that *C* is a non-decreasing function with respect to $\frac{R}{n}$. We know that $C=e^{\frac{h(Y_{1})}{n}-\frac{h(Y)}{n}}$ subject to $Y_{1}+Y_{2}=Y$ and $\frac{h(Y)}{n}-\frac{h(Y_{2})}{n}\leq \frac{R}{n}$. Thus, for a larger value of $\frac{R}{n}$, there at least exists a $\frac{h(Y_{2})}{n}$ that is non-increasing. It further leads to a non-decreasing $\frac{h(Y_{1})}{n}$. Therefore, there at least exists a C(·,R+ΔR) that is not smaller than C(·,R), ∀ΔR>0, i.e., *C* is non-decreasing with respect to $\frac{R}{n}$.

Then, we take the logarithm of $C^{n}$ and compute the partial derivative with respect to *n*:

(A6) $$\begin{array}{cc} \frac{\partial }{\partial n}n\cdot \log C\left(\frac{R}{n}\right)= & \log C\left(\frac{R}{n}\right)+\frac{n}{C}\frac{\partial }{\partial n}C\left(\frac{R}{n}\right)\\ = & \log C\left(\frac{R}{n}\right)+\frac{nC^{\prime }}{C}\cdot \left(-\frac{R}{n^{2}}\right). \end{array}$$

We know that C∈[0,1] and non-decreasing. Thus, (A6) is less than 0 when C∈(0,1). This completes the proof, where we show that $C^{n}$ is decreasing with respect to *n*.

## Appendix D. Proof of Theorem 4

Let $X^{\prime }$ be a copy of *X*. *X* can be written as a linear combination $X=tX^{\prime }+X_{2}$, where $X_{2}$ is zero-mean and independent of $X^{\prime }$, $h\left(X\right)-h\left(X_{2}\right)\leq R$, t∈[0,1]. Assume that there exists a Brenier map $Y=\nabla \varphi (X^{\prime })$. Similar to the proof of Theorem 3, this case is also in $\Pi (P_{X},P_{Y},R)$. Then, we have

(A7) $$\begin{array}{cc} & \int_{X\times Y}\nabla V\left(x\right)\cdot \left(y-x\right)\;dP\\ = & \;\int \int \;\nabla V\left(tx^{\prime }+x_{2}\right)\cdot \nabla \varphi \left(x^{\prime }\right)\;dP_{X^{\prime }}\;dP_{X_{2}}-n\\ = & \;\int \int \;\left(\nabla V\left(tx^{\prime }+x_{2}\right)-t\cdot \nabla V\left(x^{\prime }\right)\right)\cdot \nabla \varphi \left(x^{\prime }\right)\;dP_{X^{\prime }}\;dP_{X_{2}}+t\int \nabla V\left(x^{\prime }\right)\cdot \nabla \varphi \left(x^{\prime }\right)\;dP_{X^{\prime }}-n \end{array}$$

(A8) $$\begin{array}{cc} = & \;\int \int \;\left(\nabla V\left(tx^{\prime }+x_{2}\right)-t\cdot \nabla V\left(x^{\prime }\right)\right)\cdot \nabla \varphi \left(x^{\prime }\right)\;dP_{X^{\prime }}\;dP_{X_{2}}+t\int \Delta \varphi \;dP_{X^{\prime }}-n \end{array}$$

(A9) $$\begin{array}{cc} \geq & t\cdot ne^{\frac{1}{n}\left(h\left(Y\right)-h\left(X\right)\right)}-\epsilon^{\prime }-n, \end{array}$$

where we use Lemma A2 in (A7) and (A8). In (A9), we let $\epsilon^{\prime }=-\;\int \int \;\left(\nabla V\left(tx^{\prime }+x_{2}\right)-t\cdot \nabla V\left(x^{\prime }\right)\right)\cdot \nabla \varphi \left(x^{\prime }\right)\;dP_{X^{\prime }}\;dP_{X_{2}}$ and apply (A15). After changing the order of integral, we can see that $\int \nabla V\left(tx^{\prime }+x_{2}\right)\;dP_{X_{2}}$ is a smoothed version of $\nabla V(tx^{\prime })$. When ∇V is a linear function perturbed by zero-mean noise, i.e., ∇V(tx)=t·∇V(x)+W, the integral of $x_{2}$ is cancelled out and $\epsilon^{\prime }=0$. By taking $C_{x}\left(P_{X},R\right)=t$, we complete the proof.

## Appendix E. Proof of Theorem 5

According to Theorem 3, $\forall z_{0}\in Z$, there exists such $P_{X,Y|Z=z_{0}}\in \Pi \left(P_{X|Z=z_{0}},P_{Y|Z=z_{0}};R\right)$ satisfying $I(X;Y|Z=z_{0})\leq R$ and following

(A10) $$\begin{array}{cc} \; & \frac{\lambda }{2}{E[∥X-Y∥}^{2}\left|Z=z_{0}\right]\\ \leq & E\left[V\left(Y|Z=z_{0}\right)\right]-E\left[V\left(X\right)\right]+n-nC\left(P_{Y}|Z=z_{0},R\right)e^{\frac{1}{n}\left(h\left(Y|Z=z_{0}\right)-h\left(X\right)\right)}\\ \leq & E\left[V\left(Y|Z=z_{0}\right)\right]-E\left[V\left(X\right)\right]+n-nC^{\prime }\left(P_{Y|Z},R\right)e^{\frac{1}{n}\left(h\left(Y|Z=z_{0}\right)-h\left(X\right)\right)}. \end{array}$$

The conditional mutual information of the specific distribution $P_{X,Y|Z}$ can be bounded in this case as follows:

$$I\left(X;Y|Z\right)=E_{P_{Z}}\left[I\left(X;Y|Z=z_{0}\right)\right]\leq E_{P_{Z}}\left[R\right]=R.$$

Therefore, this $P_{X,Y|Z=z_{0}}$ yields the following estimate:

(A11) $$\begin{array}{cc} & \frac{\lambda }{2}W_{2}^{2}\left(P_{X|Z},P_{Y|Z}|P_{Z};R\right)\\ \leq & \frac{\lambda }{2}E_{P_{Z}}{\{E[∥X-Y∥}^{2}|Z=z_{0}]\} \end{array}$$

(A12) $$\begin{array}{cc} \leq & E\left[V\left(Y|Z\right)\right]-E\left[V\left(X\right)\right]+n-nC^{\prime }\left(P_{Y|Z},R\right)E_{P_{Z}}\left[e^{\frac{1}{n}\left(h\left(Y|Z=z_{0}\right)-h\left(X\right)\right)}\right] \end{array}$$

(A13) $$\begin{array}{cc} \leq & E\left[V\left(Y|Z\right)\right]-E\left[V\left(X\right)\right]+n-nC^{\prime }\left(P_{Y|Z},R\right)e^{\frac{1}{n}\left(E_{P_{Z}}\left[h\left(Y|Z=z_{0}\right)\right]-h\left(X\right)\right)}\\ = & E\left[V\left(Y|Z\right)\right]-E\left[V\left(X\right)\right]+n-nC^{\prime }\left(P_{Y|Z},R\right)e^{\frac{1}{n}\left(h\left(Y|Z\right)-h\left(X\right)\right)}, \end{array}$$

where (A11) follows from definition (7), (A12) follows from (A10), and (A13) follows from Jensen’s inequality. This completes the proof.

## Appendix F. Background Material

**Theorem** **A1** (Brenier’s, Theorem 2.12 [48]). *Let $P_{X}\in P\left(X\right)$, $P_{Y}\in P\left(Y\right)$ with $X\subset R^{n}$, $Y\subset R^{n}$ and assume that $dP_{X}$, $dP_{Y}$ both have finite second moments. If $P_{X}$ does not give mass to small sets, then, for the Kantorovich problem with cost $c\left(x,y\right)=\frac{1}{2}{∥x-y∥}^{2}$, ∃!φ:X→R gives the optimal coupling*

$$P^{*}={\left(Id\times \nabla \varphi \right)}_{\#}P_{X},$$

*where φ is convex. ∇φ is called the Brenier map.*

**Lemma** **A1** (Theorem 9.17 [48]). *Let $d\mu =e^{-V}$. Let $f=\frac{dP_{X}}{d\mu }$ being a Radon–Nikodym derivative between two measures $P_{X}$ and μ. Let ∇φ be a Brenier map. We have*

(A14) $$\begin{array}{cc} \int \nabla V(x)\cdot (\nabla \varphi (x)-x)f(x)d\mu (x)\geq & \int [(\Delta \varphi -n)f+(\nabla \varphi -x)\cdot \nabla f]\;d\mu \\ = & \int \Delta \varphi f\;d\mu -n+\int (\nabla \varphi -x)\cdot \nabla f\;d\mu , \end{array}$$

*where ∇φ(x)−x is called displacement. For the first term of (A14), because φ is convex, from ([4] Lemma 2.6), we have*

(A15) $$\int \Delta \varphi \;dP_{X}\geq ne^{\frac{1}{n}\left(h\left(\nabla \varphi \left(X\right)\right)-h\left(X\right)\right)}.$$

*Moreover, the last term of (A14) can be bounded using Cauchy–Schwarz inequality as follows:*

$$\begin{array}{cc} \int (\nabla \varphi -x)\cdot \nabla f\;d\mu \geq & -{\left[{\int ∥\nabla \varphi -x∥}^{2}f\;d\mu \right]}^{1/2}{\left[\int \frac{{∥\nabla f∥}^{2}}{f}d\mu \right]}^{1/2}\\ = & -W_{2}\left(P_{X},\nabla \varphi_{\#}P_{X}\right)\cdot \sqrt{I(P_{X}|\mu )}. \end{array}$$

**Lemma** **A2** (Fact 7 [3]). *For any $\nabla \varphi \in L^{1}\left(X\right)\cap L^{2}\left(X\right)$ on a Polish space (X,μ) and $d\mu =e^{-V}$, we have*

∫Δφdμ=∫∇φ·∇Vdμ.

**Definition** **A1** (Lower Semi-Continuity, Appendix B, p. 602 [49]). *Given a metric space X, f:X→R∪{∞} is lower semi-continuous if there exists a convergent sequence $\left\{x_{n}\right\},x_{n}\to x$, that $f\left(x\right)\leq \underset{n\to \infty }{lim inf}f\left(x_{n}\right)$, where $\underset{n\to \infty }{lim inf}x_{n}:=\lim_{n\to \infty }\left(\underset{m\geq n}{\inf}x_{m}\right)$.*

**Definition** **A2** (Log-Concavity, Definition 2.1 [50]). *A density function f with respect to the Lebesgue measure on $\left(R^{n},B^{n}\right)$ is log-concave if $f=e^{-V}$, where V is a convex function.*

**Definition** **A3** (Strong Log-Concavity, Definition 2.8 [50]). *A density function f is called strongly log-concave if it has the form*

f(x)=g(x)φ(x),

*with some log-concave g and some φ∼N(μ,Σ).*

## References

[1] Talagrand M. (1996). Transportation cost for Gaussian and other product measures. Geom. Funct. Anal..

[2] Bakry D., Bolley F., Gentil I. (2012). Dimension dependent hypercontractivity for Gaussian kernels. Probab. Theory Relat. Fields.

[3] Cordero-Erausquin D. (2017). Transport inequalities for log-concave measures, quantitative forms, and applications. Can. J. Math..

[4] Bolley F., Gentil I., Guillin A. (2018). Dimensional improvements of the logarithmic Sobolev, Talagrand and Brascamp–Lieb inequalities. Ann. Probab..

[5] Raginsky M., Sason I. (2018). Concentration of Measure Inequalities in Information Theory, Communications and Coding. Foundations and Trends in Communications and Information Theory.

[6] Zhang R., Chen C., Li C., Carin L. Policy Optimization as Wasserstein Gradient Flows. Proceedings of the International Conference on Machine Learning.

[7] Montavon G., Müller K.R., Cuturi M. Wasserstein Training of Restricted Boltzmann Machines. Proceedings of the 30th International Conference on Neural Information Processing Systems.

[8] Arjovsky M., Chintala S., Bottou L. Wasserstein Generative Adversarial Networks. Proceedings of the International Conference on Machine Learning.

[9] Rigollet P., Weed J. (2019). Uncoupled isotonic regression via minimum Wasserstein deconvolution. Inf. Inference.

[10] Cuturi M. (2013). Sinkhorn Distances: Lightspeed Computation of Optimal Transportation Distances. Adv. Neural Inf. Process. Syst..

[11] Wang S., Stavrou P.A., Skoglund M. Generalized Talagrand Inequality for Sinkhorn Distance using Entropy Power Inequality. Proceedings of the 2021 IEEE Information Theory Workshop (ITW).

[12] Benamou J.D., Brenier Y. (2000). A computational fluid mechanics solution to the Monge-Kantorovich mass transfer problem. Numer. Math..

[13] Villani C. (2008). Optimal Transport: Old and New.

[14] Schrödinger E. (1931). Über die Umkehrung der Naturgesetze.

[15] Léonard C. (2014). A survey of the Schrödinger problem and some of its connections with optimal transport. Discret. Contin. Dyn. Syst..

[16] Chen Y., Georgiou T.T., Pavon M. (2016). On the relation between optimal transport and Schrödinger bridges: A stochastic control viewpoint. J. Optim.Theory Appl..

[17] Chen Y., Georgiou T.T., Pavon M. (2016). Optimal transport over a linear dynamical system. IEEE Trans. Autom. Control.

[18] Conforti G. (2019). A second order equation for Schrödinger bridges with applications to the hot gas experiment and entropic transportation cost. Probab. Theory Relat. Fields.

[19] Conforti G., Ripani L. (2020). Around the entropic Talagrand inequality. Bernoulli.

[20] Bai Y., Wu X., Özgür A. Information Constrained Optimal Transport: From Talagrand, to Marton, to Cover. Proceedings of the IEEE International Symposium on Information Theory (ISIT).

[21] Rigollet P., Weed J. (2018). Entropic optimal transport is maximum-likelihood deconvolution. C. R. Mathem..

[22] Mena G., Niles-Weed J. (2019). Statistical bounds for entropic optimal transport: Sample complexity and the central limit theorem. Advances in Neural Information Processing Systems.

[23] Genevay A., Chizat L., Bach F., Cuturi M., Peyré G. Sample Complexity of Sinkhorn Divergences. Proceedings of the Twenty-Second International Conference on Artificial Intelligence and Statistics.

[24] Reshetova D., Bai Y., Wu X., Özgür A. Understanding Entropic Regularization in GANs. Proceedings of the IEEE International Symposium on Information Theory (ISIT).

[25] Shannon C.E. (1948). A mathematical theory of communication. Bell Syst. Tech. J..

[26] Stam A.J. (1959). Some inequalities satisfied by the quantities of information of Fisher and Shannon. Inf. Control.

[27] Rioul O. (2010). Information theoretic proofs of entropy power inequalities. IEEE Trans. Inf. Theory.

[28] Courtade T.A., Fathi M., Pananjady A. (2018). Quantitative stability of the entropy power inequality. IEEE Trans. Inf. Theory.

[29] Bobkov S., Madiman M. (2012). Reverse Brunn—Minkowski and reverse entropy power inequalities for convex measures. J. Funct. Anal..

[30] Bobkov S.G., Madiman M.M. (2013). On the problem of reversibility of the entropy power inequality. Limit Theorems in Probability, Statistics and Number Theory.

[31] Courtade T.A. (2017). A strong entropy power inequality. IEEE Trans. Inf. Theory.

[32] Cover T.M. (1999). Elements of Information Theory.

[33] Tamanini L. (2020). A generalization of Costa’s Entropy Power Inequality. arXiv.

[34] Monge G. (1781). Mémoire sur la théorie des déblais et des remblais. Histoire de l’Académie Royale des Sciences de Paris.

[35] Kantorovich L.V. (2006). On the translocation of masses. J. Math. Sci..

[36] Kantorovich L.V. (2006). On a Problem of Monge. J. Math. Sci..

[37] Dupuis P., Ellis R.S. (2011). A Weak Convergence Approach to the Theory of Large Deviations.

[38] Luenberger D.G. (1997). Optimization by Vector Space Methods.

[39] Blower G. (2003). The Gaussian isoperimetric inequality and transportation. Positivity.

[40] Otto F., Villani C. (2000). Generalization of an inequality by Talagrand and links with the logarithmic Sobolev inequality. J. Funct. Anal..

[41] Bakry D., Ledoux M. (2006). A logarithmic Sobolev form of the Li-Yau parabolic inequality. Rev. Matemática Iberoam..

[42] Masry E. (1991). Multivariate probability density deconvolution for stationary random processes. IEEE Trans. Inf. Theory.

[43] Stefanski L.A., Carroll R.J. (1990). Deconvolving kernel density estimators. Statistics.

[44] Fan J. (1991). On the optimal rates of convergence for nonparametric deconvolution problems. Ann. Stat..

[45] Janati H., Muzellec B., Peyré G., Cuturi M. (2020). Entropic optimal transport between unbalanced Gaussian measures has a closed form. Adv. Neural Inf. Process. Syst..

[46] Marton K. (1996). A measure concentration inequality for contracting Markov chains. Geom. Funct. Anal..

[47] Flamary R., Courty N., Gramfort A., Alaya M.Z., Boisbunon A., Chambon S., Chapel L., Corenflos A., Fatras K., Fournier N. (2021). POT: Python Optimal Transport. J. Mach. Learn. Res..

[48] Villani C. (2003). Topics in Optimal Transportation.

[49] Puterman M.L. (2014). Markov Decision Processes: Discrete Stochastic Dynamic Programming.

[50] Saumard A., Wellner J.A. (2014). Log-concavity and strong log-concavity: A review. Stat. Surv..

